# Development and Evaluation of a Smartphone App-Based Rapid 25-Hydroxy Vitamin D Test

**DOI:** 10.3390/diagnostics15222916

**Published:** 2025-11-18

**Authors:** SoYeong Han, Seung Hyun Kim, MyungJin Kim, NaMi Park, Junnan Gu, Sun Jong Kim, Suk Yong Lee, Jeongku Seo

**Affiliations:** 1 BioFront, HangGang Xi Tower, 401, Yangcheon-ro, Gangseo-Gu, Seoul-si 07258, Republic of Korea; syhan@biofront.co.kr (S.H.); biomjkim@biofront.co.kr (M.K.); pnm@biofront.co.kr (N.P.); tracy@biofront.co.kr (J.G.); naturekim71@biofront.co.kr (S.J.K.); 2 DiaVision, Startup Room 5, BI Center, 2F Healthcare Innovation Park, SNUBH, 172, Dolma-ro, Bundang-Gu, Seongnam-si 13605, Republic of Korea; sh0510@diavision.co.kr

**Keywords:** smartphone-based diagnostics, point-of-care testing, 25-hydroxyvitamin D, semi-quantitative immunoassay

## Abstract

**Objectives:** The purpose of this study is to develop and verify a sandwich-type lateral flow immunoassay (LFA) integrated with a smartphone, enabling semi-quantitative 25-hydroxyvitamin D [25(OH)D] measurement including automated image analysis function, thereby establishing a reliable and accessible vitamin D evaluation system for point-of-care (POCT). **Methods**: A smartphone-based sandwich-type LFA was constructed, and 25(OH)D was measured semi-quantitatively. The system combined a customized test strip with an automatic image acquisition, calibration, and classification module integrated into an application dedicated to a smartphone. Analysis performance, reproducibility, and equivalence between sample types were comprehensively evaluated. **Results**: The developed analysis achieved a detection range of 5–100 ng/mL, and there were little interference and cross-reactivity for endogenous substances or structurally similar vitamin D derivatives. The image processing algorithm accurately classified the samples into three clinically important categories: deficiency (<20 ng/mL), insufficient (20–30 ng/mL), and sufficient (>30 ng/mL). Cross-platform testing between Android and iOS devices showed excellent reproducibility (r = 0.99, R^2^ = 0.9967). Comparative analysis with the Atellica IM 1600 analyzer showed a high degree of agreement between 97.0% category consensus and κ = 0.951 (r = 0.99, R^2^ ≥ 0.98). Comparative tests between serum and capillary samples also confirmed a 100% classification agreement rate and an overall diagnostic accuracy of 95.5%. **Conclusions**: This next-generation smartphone integration platform enables rapid, accurate, and semi-quantitative detection of 25(OH)D from fingerstick and serum specimens. By combining the sandwich-type LFA design with computational-based imaging analysis, the system effectively overcomes the major limitations of small-molecule immunoassay and has the potential to be applied to field diagnosis (POCT), decentralized diagnostics, and vitamin D screening in large populations.

## 1. Introduction

Vitamin D is known to play an important role in maintaining calcium and phosphorus homeostasis [1], regulating bone matrix and bone remodeling [2], and regulating innate and acquired immune responses [3,4]. Various recent studies have reported that vitamin D is involved not only in bone related functions, but also in various physiological and pathological processes such as glucose metabolism, cardiovascular regulation, and immune regulation [5,6,7,8]. Vitamin D deficiency is associated with an increased risk of developing multiple diseases such as osteoporosis, adult osteomalacia, pediatric rickets, type 2 diabetes, hypertension, cardiovascular disease, autoimmune disease, infectious disease, colon cancer, and breast cancer [9,10,11,12]. These various effects suggest that vitamin D goes beyond simple nutrients to act as a multifunctional physiological regulator like hormones and imply that it may play an important role throughout human health.

Epidemiologic data from around the world suggest that vitamin D deficiency and deficiency status are still widespread, with more than 1 billion people estimated to be affected worldwide [13]. High deficiency rates have been reported in the elderly, people with limited sunlight exposure, people with dark skin color, people with malabsorption, and residents of high-level regions. Therefore, regular monitoring of vitamin D is essential for maintaining physiological balance and preventing disease. Clinically, serum 25-hydroxyvitamin D [25(OH)D], which reflects both dietary intake and endogenous synthesis, is used as the most reliable indicator for the assessment of vitamin D status. Although exact clinical criteria for vitamin D have not yet been established, it has been reported that vitamin D concentrations, in general, can be classified as deficient (<20 ng/mL), insufficient (20–30 ng/mL), and sufficient (>30 ng/mL) [14,15]. Particularly for severe deficiency, the increase of vitamin D may be limited with general supplementation therapy due to differences in an individual’s absorption rate or metabolic resistance, and excessive supplementation may cause hypercalcemia or nephrotoxicity [16]. Therefore, it is important to accurately diagnose vitamin D deficiency early and enable proper therapeutic intervention and prevention of side effects.

Chemiluminescence Immunoassay (CLIA), enzyme-linked immunosorbent assay (ELISA), and liquid chromatography–tandem mass spectrometry (LC-MS/MS) are widely used as standard test methods for quantitative analysis of 25(OH)D [17]. These methods provide high precision and sensitivity, but they have the disadvantage of requiring central laboratory infrastructure, expensive equipment, skilled personnel, and long and expensive testing. Due to these constraints, they are not suitable for repeated vitamin D monitoring or point-of-care test (POCT) tests in resource limited environments [18,19,20]. To overcome these shortcomings, a lateral flow assay (LFA) vitamin D measurement method that enables simple and low-cost rapid field testing is attracting attention as an alternative. However, the existing LFA-based vitamin D assay has several limitations. Since 25(OH)D has a small molecular size, two antibodies cannot recognize it at the same time, so the existing LFA has used a competitive analysis method, but this principle of action has limitations in sensitivity and reproducibility

Therefore, it is necessary to develop a user-friendly field diagnosis (POCT) system capable of continuous vitamin D monitoring that is reliable and exhibits quantitative accuracy. Recently, in addition to precision-based examination, the demand for POCT technology that provides rapid results in the field is increasing. Currently, most vitamin D POCT equipment on the market is used in hospitals by patients visiting the hospital, and separate measurement equipment is required (Supplementary Table S1). To meet these clinical requirements, in this study, it was intended to develop a kit that semi-quantitatively measures vitamin D concentration within about 15 min using fingerstick blood or serum, can be quickly classified into three stages of deficiency, insufficient, and sufficient, and shows accuracy like that of the existing vitamin D analyzer. In addition, by performing quantitative image analysis through a smartphone-based application, objectivity and reproducibility were improved compared to visual reading. The Vita-D Rapid Kit can be easily used in a clinical environment or at home without additional analysis equipment, so it is a next-generation vitamin D diagnostic platform that combines user convenience and accessibility. We have developed a new sandwich-type LFA using an anti-idiotype antibody. The system enables stable sandwich complex formation and improves signal sensitivity by using an anti-idiotype antibody that specifically recognizes the structural change that occurs when 25(OH)D binds to the capture antibody. In addition, the accuracy and reproducibility of the analysis were improved by inhibiting non-specific adsorption.

Advances in mobile health technology (mHealth) and smartphone image analysis have opened new possibilities for decentralized diagnostic systems [21,22,23]. Modern smartphones are equipped with high-resolution cameras, high-performance processors, wireless connectivity, enabling precise image acquisition, real-time data processing, and cloud integration. Several recent studies have reported that self-health monitoring in the elderly [24], smartphone-based POCT system for measuring HbA1c in diabetic patients [25], atrial fibrillation (AF) large-scale population screening, and high-sensitivity POCT platform for detecting myocardial injury markers (cTnI) [26]. In addition, a smartphone application was developed to track female reproductive hormones (LH, hCG, etc.), demonstrating the potential to extend beyond short-term ovulation prediction to long-term hormone monitoring and women’s health care [27,28]. These trends suggest that the smartphone-based in vitro diagnostic (IVD) image analysis application presented in this study has the potential to be practical in various clinical and public health fields, including self-diagnosis, chronic disease monitoring, and large-scale screening for the elderly. These features collectively transform traditional testing into a continuous, data-driven health management ecosystem [29,30,31]. Using this principle, research and development of kits for SARS-CoV-2 antigens have been reported [32], and our group also developed a smartphone-linked lateral flow platform to prove the possibility of combining optical signal processing and quantitative immunoassay design to produce neutralizing antibodies after various COVID vaccines injection [33].

The integration of advanced POCT technologies with smartphone-assisted imaging has enabled the development of next-generation diagnostic systems that combine immunochromatographic assay principles with digital signal processing. Smartphone-linked LFAs can utilize algorithm-driven analysis to achieve semi-quantitative or fully quantitative readouts, even from minimally invasive samples such as fingerstick blood. The integration of mobile applications further allows automated calibration, objective interpretation, and real-time signal quantification. Beyond immediate measurement, smartphone connectivity enables secure data storage, longitudinal tracking of individual vitamin D trends, and cloud-based data sharing with healthcare providers facilitating remote monitoring and personalized nutritional management.

In this study, the development process, analysis performance evaluation, and clinical verification results of the Vita-D Lapid Kit were described. The performance of serum and capillary blood samples was evaluated by comparing with the existing laboratory standard assay method, and the ability to distinguish between deficiencies (<20 ng/mL) in clinical significance, reproducibility, stability, and ease of use in real environments were verified. Finally, the possibility of integration within the digital health ecosystem aimed at prevention and personalized medicine and the future implications of decentralized vitamin D monitoring were discussed.

## 2. Materials and Methods

### 2.1. Development of the Smartphone-Based Vitamin D Rapid Assay

The Vita-D Rapid Kit is an in vitro diagnostic platform for semi-quantitative detection of total 25(OH)D in capillary blood or serum. Using a sandwich type LFIA format, the assay classifies vitamin D status as deficiency, insufficient, or sufficient at the point of care.

The sandwich-type LFA developed in this study was designed based on an anti-idiotype recognition mechanism and optimized buffer composition, comprising a control line (C) coated with anti-chicken IgY antibodies. The capture antibody, a sheep monoclonal antibody specific to 25(OH)D, was passively adsorbed onto colloidal gold nanoparticles (AuNPs, 40 nm) and subsequently blocked with 10% BSA for stabilization and then centrifuge at 10,000 rpm, for 10 min, discard the supernatant. The detection antibody is a sheep anti-idiotype antibody generated against the 25(OH)D-Ab complex (10mM PBS, 2% Sucrose, pH7.4), and it was directionally immobilized onto the nitrocellulose membrane via Protein A/G to ensure proper orientation. When 25(OH)D is present in the sample, immune complexes form and bind to the T line, producing a visible red band proportional to the analyte concentration. The reaction buffer was optimized using a Tris–HCl–based formulation containing MES hydrate, Tween 20, casein, and BSA to enhance assay performance. Following the reaction, results are acquired and interpreted by a dedicated smartphone application (Supplementary Figure S1). The intensity of the signal is proportional to the 25(OH)D concentration, allowing for semi-quantitative interpretation.

### 2.2. Image-Based Quantification Using Smartphone Application

Our system employs a server-side AI inference structure where the app extracts Region of interest (ROI) from captured images, performs perspective/rotation correction and white balance adjustment, then transmits only anonymized ROI patches (not full original images) via TLS 1.3 encryption to the server. The server operates as a containerized stateless inference microservice running a two-stage AI pipeline: (1) A lightweight Convolutional neural network (CNN) backbone-based single-pass detector precisely localizes the strip region and computes its position, orientation, and scale. (2) A segmentation model combining a lightweight CNN backbone with SE-attention modules, optimized through quantization and acceleration, performs low-latency C/T line segmentation. The server returns line masks, computed T/C ratios, and QC flags (e.g., overexposure, insufficient reaction). The app then applies calibration regression to determine final vitamin D concentration and deficiency grade. Transmitted images are not stored by default (anonymized QC logging only with user consent). Each result is tagged with model version and calibration version for full traceability.

Images of the test strip were captured using smartphone cameras (minimum OS requirements: Android 8.0 or later, iOS 15 or later). A flash-control algorithm was implemented to standardize illumination, while focus-guidance software ensured optimal image acquisition. Captured images were converted into color spectrum data, and pixel intensities for both the test (T) and control (C) lines were extracted.

To achieve standardized quantification, rather than directly using the relative intensity ratio (IT/IC), the system measured the pixel intensities of five reference lines (Ik, k = 1, …, 5) printed on the strip background, each corresponding to a predefined nominal reference signal (Rk). These (Ik, Rk) pairs were used to construct a multi-point calibration curve, mapping raw pixel intensity to standardized signal values. A least-squares regression was applied to derive the calibration function:

S = g(I) = a_0_ + a_1_I (+ a_2_I^2^)

where I denote the measured pixel intensity, and Rk represents the nominal signal level of each reference line. Local background correction and outlier removal were performed to minimize variability due to device and illumination differences.

Using this calibration function, the raw intensities of the test and control lines (I_T_, I_C_), were transformed into standardized signal values:

*S_T_* = *g*(*I_T_*), *S_C_* = *g*(*I_C_*)

For improved robustness, a normalized signal was calculated as:

$$S_{T|C}=\frac{s_{T}}{s_{c}}$$

The final vitamin D concentration was then estimated using a regression model derived from independent reference samples:

Vitamin D (ng/mL) = *αS_T_* + *β*

or, when using the control-normalized signal,

Vitamin D (ng/mL) = α′S_T∣C_ + β′

Here, *α*, *β* (or *α*′, *β*′) are calibration coefficients determined from standard curves generated with reference materials. This calibration-based approach compensates for device-to-device variability and illumination inconsistencies, ensuring that T and C line signals are normalized to an absolute or quasi-absolute scale prior to concentration estimation.

### 2.3. Evaluation of Reproducibility and Repeatability

Reproducibility and repeatability were assessed using Android (Galaxy S22) and iOS (iPhone 12 Pro Max) devices. A single operator performed five replicate measurements for each reference material-negative standard (vitamin D depleted serum, VITDSC-N, Molecular Depot, Cat#S2010003, San Diego, CA, USA), low-level positive standard (VITDSC-L, DEQAS, Cat#618, London, UK), medium-level positive standard (VITDSC-M, DEQAS, Cat#577, Lonon, UK), and high-level positive standard (VITDSC-H, DEQAS, Cat#600, London, UK)—across three production lots. Additionally, three independent operators each performed five replicate measurements using a single lot. Site-to-site reproducibility was further assessed by testing one lot at three separate locations (Supplementary Tables S2 and S3).

### 2.4. Evaluation of Dynamic Range and Limit of Detection (LoD)

To prepare samples with constant Vitamin D concentrations, the Vitamin D standard material, Liquicheck^TM^ Specialty Immunoassay Control (Bio-rad, Cat#359, Irvine, CA, USA), was mixed with Vitamin D Depleted Serum to produce eight dilution levels. The Total Vitamin D concentrations of these samples were quantified using the Atellica IM 1600 Analyzer (Atellica IM Vitamin D Total, Siemens Healthcare Diagnostics, Cat#10995719, Eschborn, Germany). and each sample was measured in triplicate. The measurement range and the Limit of Detection (LoD) of the Vita-D kit was evaluated using the eight prepared concentration samples. Each concentration level was tested for five consecutive days with five replicates per day, and the LoD was defined as the lowest concentration at which both the visual identification of the test line and the app-based quantitative measurement were consistently detectable. Subsequently, 20 additional replicate tests were conducted over three days within the determined concentration range, and the final LoD was confirmed when the signal was detectable in more than 90% of the tests and showed more than 95% consistency (Supplementary Table S4). In addition, the LoD was cross-validated using the statistical approach recommended by the CLSI EP17-A2 guideline, where the limit of blank (LoB) was first estimated from 20 blank replicates (LoB = mean_blank + 1.645 × SD_blank), and the LoD was calculated as LoD = LoB + 1.645 × SD_low using 20 vitamin D depleted serum replicates.

### 2.5. Cross-Reactivity and Interference Assessment

To evaluate potential cross-reactivity and interference effects were evaluated using the same standard materials for reproducibility and repeatability assessments. Each standard was spiked with predefined concentrations of potential interfering or cross-reactive substances, as listed in Supplementary Table S5, to simulate clinically relevant conditions. All spiked samples were independently prepared in triplicate, and assay performance was subsequently analyzed to identify any significant deviations in signal response attributable to cross-reactivity or interference.

### 2.6. Correlation with a Commercial Vitamin D Analyzer

Residual serum specimens (*n* = 100) were obtained under institutional review board (IRB) approval (IRB No. 2023-RR04) and analyzed for total 25(OH)D concentrations using the Atellica IM 1600 Analyzer. The samples were classified according to the manufacturer’s clinical interpretation criteria as deficient (<20 ng/mL), insufficient (20–30 ng/mL), or sufficient (>30–100 ng/mL). Categorical agreement between the Vita-D Rapid Kit and the reference analyzer was then evaluated based on these classifications.

### 2.7. Specimen Type Equivalence

The equivalence between capillary blood and serum samples was evaluated under institutional review board (IRB) approval (IRB No. P01-202310-02-012). Paired specimens (*n* = 22) were obtained from participants with informed consent, and both sample types were analyzed using the same procedures described above. According to the kit instructions, 10 μL of capillary blood and 5 μL of serum were used for testing, and identical assay procedures were applied for both sample types.

### 2.8. Statistical Analysis

Agreement between the Vita-D Rapid Kit and the reference method was assessed using overall percent agreement and Cohen’s kappa (κ) statistics. Weighted κ values were calculated to account for the ordinal nature of the categorical outcomes. For continuous variables, 95% confidence intervals (CIs) were determined using Welch’s *t*-test, while the Wilson score method was applied to estimate CIs for proportions. The correlation between the two continuous variables was evaluated by Pearson correlation coefficient (r), and explanatory power was expressed as R^2^.

## 3. Results

### 3.1. System Architecture and Analytical Design

The Vita-D Rapid Kit integrates a custom LFIA strip with a dedicated smartphone application to enable semi-quantitative detection of 25(OH)D. The C line serves as an internal reference, allowing the built-in image processing algorithm to normalize signals across different devices.

The developed system presents a novel immunorecognition strategy for small molecules, overcoming the inherent limitations of conventional competitive LFAs. In this study, an anti-idiotype antibody was developed to specifically recognize the antigen–antibody binding site of the 25(OH)D complex. Consequently, the detection antibody recognizes the 25(OH)D–Ab complex as a new target, effectively overcoming steric hindrance while achieving high specificity and signal linearity (Supplementary Figure S1). In addition, optimization of the reaction buffer composition helped maintain the stability of biologically active 25(OH)D and promote its appropriate dissociation, thereby improving antibody binding efficiency. The buffer also reduced nonspecific adsorption of the lipophilic analyte, resulting in high reproducibility under various sample conditions, including serum and nasal fluid. Compared to conventional competitive LFAs, the proposed system demonstrated superior performance in terms of linearity, signal-to-noise ratio (S/N), and reproducibility.

**Figure 1:**
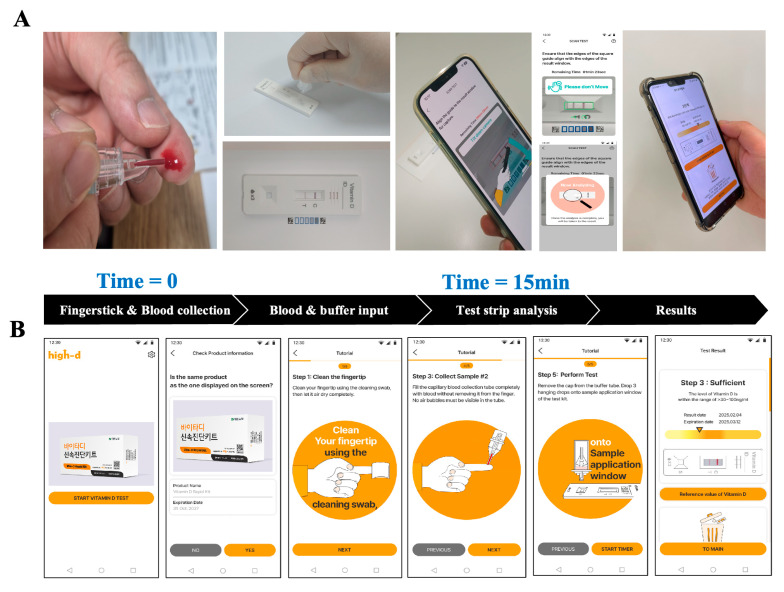
Workflow of the Vita-D Rapid Kit using the high-d application. (**A**) Schematic illustration of the 25(OH)D measurement process using capillary blood and the smartphone-based high-d application. (**B**) Representative user interface screenshots showing step-by-step guidance during sample testing and result interpretation.

Figure 1 illustrates the overall configuration and operational workflow of the Vita-D Rapid Kit platform. As shown in Figure 1A, the assay begins with sample loading onto the LFIA strip, followed by capillary driven migration, target–antibody binding, and signal generation at the test and control lines. This streamlined workflow is optimized for rapid, user-friendly operation and enables semi-quantitative detection of 25(OH)D at the point of care. Figure 1B shows a representative screenshot of the smartphone application interface during the assay. The application guides the user through each procedural step, captures the test strip image, and automatically analyzes signal intensity to provide a semi-quantitative readout. Importantly, the Vita-D Rapid Kit exhibited no evidence of cross-reactivity or interference from structurally related compounds or components, indicating high analytical specificity (Supplementary Table S5). These results demonstrate the integrated nature of the platform, which combines simple assay handling with automated data analysis to ensure reliable and specific vitamin D detection.

### 3.2. Semi-Quantitative Detection of 25(OH)D via Smartphone-Based Image Analysis

**Figure 2:**
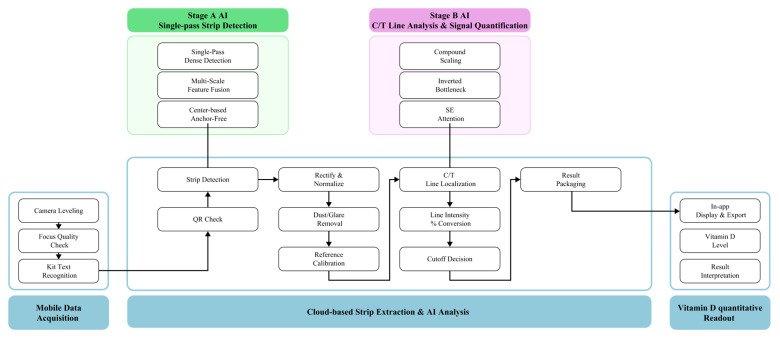
Overall workflow of the AI-powered vitamin D measurement solution. The system integrates mobile-based image acquisition, cloud-based test strip detection, AI-driven signal analysis, and quantitative result interpretation. This enables automated detection, real-time signal processing, and semi-quantitative determination of vitamin D levels from LFIA test strips.

**Figure 3:**
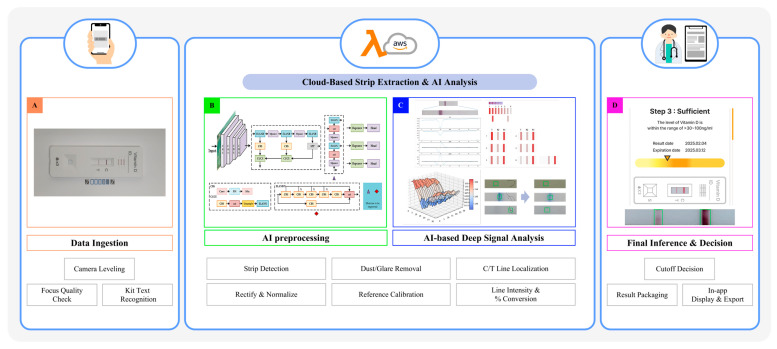
Cloud-based AI workflow for quantitative vitamin D measurement. (**A**) Data ingestion: Mobile image captured with camera leveling, focus quality control, and kit recognition. (**B**) AI preprocessing: Automated strip detection, rectification, normalization, dust/glare removal, and reference calibration. (**C**) Deep signal analysis: C/T line localization, pixel intensity quantification, and percentage conversion for accurate vitamin D signal interpretation. (**D**) Final inference and decision-making: Cutoff classification, result packaging, and in-app result display/export.

The detection signal at the T line is generated by AuNP-labeled immunocomplexes, as illustrated in Supplementary Figure S1. Upon sample application, 25(OH)D molecules bind to AuNP-conjugated capture antibodies and are subsequently immobilized by secondary antibodies coated on the nitrocellulose membrane. Accumulation of these AuNP–antibody complexes produce a colorimetric signal proportional to the analyte concentration. The smartphone application processes the optical signal through a multi-step computational workflow; (1) Automated identification of T and C line regions using edge-detection and pattern-recognition algorithms. (2) Background noise reduction and signal normalization based on C line intensity. (3) Conversion of pixel intensity values into semi-quantitative concentration levels using a pre-calibrated regression model. (4) Classification of results into clinically relevant categories: deficient (<20 ng/mL), insufficient (20–30 ng/mL), or sufficient (>30 ng/mL). This algorithmic pipeline ensures robust analytical performance and cross-device compatibility. Building upon this principle, the proposed system integrates mobile-based data acquisition with cloud-based AI analysis to achieve reproducible quantification from LFIA strips (Figure 2). During image acquisition, the application optimizes imaging conditions through camera leveling, focus assessment, and kit recognition. “Stage A” AI performs single-pass strip detection using dense detection, multi-scale feature fusion, and anchor-free localization. “Stage B” AI subsequently conducts C/T line analysis and signal quantification with compound scaling and squeeze-and-excitation (SE) attention mechanisms. Extracted signals are normalized, corrected for artifacts such as dust or glare, and calibrated before conversion into quantitative outputs. Results are displayed within the app as semi-quantitative vitamin D levels with clinical interpretation. Figure 3 demonstrates the functional workflow. Figure 3A shows guided image capture. Figure 3B depicts AI-based preprocessing, including strip detection, rectification, and normalization. Figure 3C presents deep signal analysis, where C/T regions are localized, pixel intensities quantified, and calibration applied. Finally, Figure 3D shows the inference stage, where signals are converted into clinically interpretable results. Together, these findings demonstrate that integrating AuNP-based immunocomplex detection, smartphone-based image acquisition, and cloud-based AI analysis enables accurate, device-independent quantification of vitamin D.

### 3.3. Analytical Sensitivity and Cross-Platform Performance

**Figure 4:**
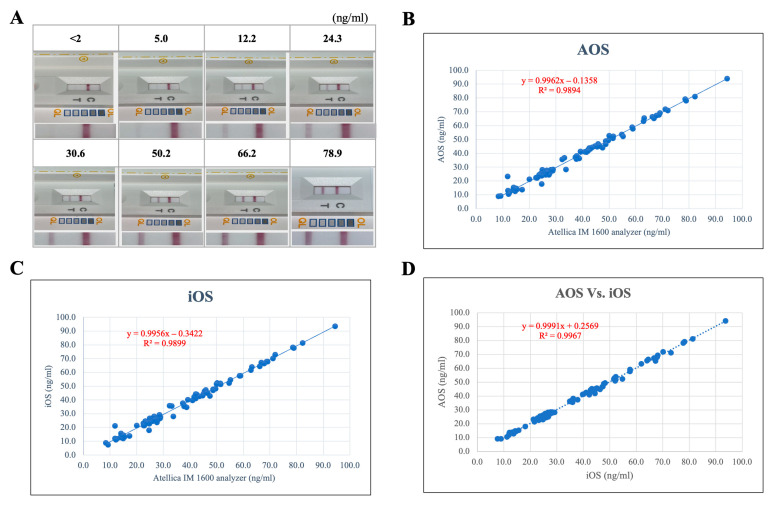
Analytical correlation and representative results from the Vita-D Rapid Kit across various vitamin D concentrations and smartphone platforms. (**A**) Representative images of Vita-D Rapid Kit test strips at different 25(OH)D concentration levels. (**B**) Correlation analysis between serum results obtained using the Atellica IM 1600 analyzer and those from the Vita-D Rapid Kit on Android devices (AOS). (**C**) Correlation between the analyzer and Vita-D Rapid Kit results on iOS devices. (**D**) Correlation between Android and iOS platforms demonstrating cross-platform consistency of the Vita-D Rapid Kit.

Representative test strip images obtained using the Vita-D Rapid Kit exhibited distinct test line intensities corresponding to varying 25(OH)D concentrations, allowing clear semi-quantitative differentiation across clinically relevant thresholds. Based on the results obtained from five replicate tests conducted over five consecutive days using the Vitamin D Rapid Kit with eight prepared concentration levels. Additional 20 repeated tests performed over three days near the lowest detectable concentration confirmed the limit of detection (LoD) to be 5 ng/mL and the signal saturation was observed in samples with concentrations above 100 ng/mL. Based on the statistical approach, the limit (LoB) of the blank was calculated as 0.83 ng/mL (mean blank value = 0.5 ng/mL, standard deviation SD_blank = 0.2 ng/mL), and the standard deviation (SD_low) of the low concentration sample was 2.5 ng/mL. The calculated detection limit (LoD) was found to be 5.0 ng/mL. Therefore, the measurement range of the Vita-D Rapid Kit was determined to be 5–100 ng/mL (Figure 4A, Supplementary Table S4). Quantitative evaluation demonstrated a strong and statistically significant correlation between the Vita-D Rapid Kit and the reference Atellica IM 1600 Analyzer across both Android (r = 0.99, R^2^ = 0.9894) (Figure 4B) and iOS (r = 0.99, R^2^ = 0.9899) (Figure 4C) platforms. Moreover, comparative analysis between Android- and iOS-derived results showed a high degree of concordance with minimal variation (r = 0.99, R^2^ = 0.9967) (Figure 4D), confirming consistent and reproducible assay performance irrespective of the operating system. Collectively, these results demonstrate the robustness of the developed platform and its suitability as a point-of-care testing (POCT) tool for reliable vitamin D monitoring.

### 3.4. Cross-Platform Analytical Reproducibility

We designated the iOS imaging/processing pipeline as a platform reference (“gold standard” for cross-platform alignment) and calibrated Android images to this reference before analysis. Specifically, we learned an Android → iOS photometric transform from paired captures of the same strips and applied it to Android ROIs prior to segmentation and quantification. The transform comprises: (i) sRGB gamma linearization, (ii) neutralzone white-balance normalization on the cassette, (iii) polynomial shading correction, and (iv) a 3 × 3 color correction matrix with intensity scaling that maps Android RGB responses onto the iOS reference space. After alignment, Android category calls reproduced the iOS decisions across the paired test set.

To evaluate cross-platform reproducibility, three independent production lots of the Vita-D Rapid Kit were tested using two major mobile operating systems: Android (Galaxy S22) and iOS (iPhone 12 Pro Max). As shown in Table 1, semi-quantitative classifications of all reference standards were consistent across both platforms, demonstrating excellent intra- and inter-device reproducibility. These results indicate that the software’s image acquisition and analysis modules are minimally influenced by hardware or operating system differences. In addition, tests performed on other smartphone models showed comparable analytical performance, further confirming the robustness of the system across various devices.

### 3.5. Comparative Analytical Accuracy with a Commercial Vitamin D Assay

Using 100 serum specimens, the Vita-D Rapid Kit demonstrated strong concordance with the Atellica IM 1600 Analyzer. Correlation analysis revealed a robust linear relationship between the two methods. Samples were categorized according to concentration, sex, and app-based interpretation levels as determined by the reference analyzer (Table 2). Categorical agreement was 93.3% (95% CI: 70.2–98.8%) for deficient samples (<20 ng/mL), 97.4% (95% CI: 86.8–99.6%) for insufficient samples (20–30 ng/mL), and 97.8% (95% CI: 88.7–99.6%) for sufficient samples (>30–100 ng/mL). The overall categorical agreement was 97.0% (95% CI: 93.0–99.1%). Semi-quantitative classification by the smartphone application showed substantial inter-method agreement (κ = 0.951). No statistically significant differences were observed between results obtained using the two software applications (Table 3). Additionally, neither sex nor age was associated with differences in classification outcomes. Collectively, these findings indicate that the Vita-D Rapid Kit provides clinical performance comparable to that of the laboratory-based reference analyzer.

### 3.6. Matrix Equivalence Between Capillary Blood and Serum

To assess matrix equivalence, paired capillary blood and serum samples were collected from the same donors and analyzed using the Vita-D Rapid Kit. Reference concentrations determined by the Atellica IM 1600 Analyzer were used as the comparator standard. Classification concordance between capillary blood and serum samples was 100% across all concentration categories (Table 4). In total, 22 paired samples were analyzed, yielding categorical agreement rates of 100% (95% CI: 75.8–100%) for deficient samples (<20 ng/mL), 86.0% (95% CI: 48.7–97.4%) for insufficient samples (20–30 ng/mL), and 100% (95% CI: 43.9–100%) for sufficient samples (>30 ng/mL), resulting in an overall accuracy of 95.5% (95% CI: 78.2–99.1%). Results obtained from Android and iOS applications were comparable, with no statistically significant differences observed between platforms (Table 5). Minor discrepancies fell within the expected analytical variability of the Atellica IM reference method. Consistent with serum testing, neither sex nor age showed any association with classification outcomes. Collectively, these findings demonstrate that capillary blood is analytically equivalent to serum for use with the Vita-D Rapid Kit, supporting its applicability for point-of-care testing (POCT) and decentralized clinical implementation.

## 4. Discussions

In this study, we presented the development and comprehensive verification results of the Vita-D Rapid Kit, a smartphone-based lateral flow immunoassay (LFIA) platform designed for semi-quantitative detection of 25-hydroxyvitamin D [25(OH)D]. By integrating advanced algorithm-based image analysis technology, this platform effectively overcomes the major limitations of existing small molecule immunoassay methods and provides a diagnostic solution suitable for point-of-care testing (POCT) environments. The rapid development of diagnostic technology integrated with smartphones has revolutionized the existing testing environment, enabling the detection of low-cost, high-neighborhood biomarkers even outside the traditional laboratory [31,34].

A persistent challenge in developing POCT systems for low-molecular weight analytes is to achieve both high analytical sensitivity and specificity [35]. Conventional immunoassays for small molecules generally adopt competitive or inhibition-based formats, since the structural constraints of small molecules make it difficult for two antibodies to bind simultaneously. Although these formats are simple and robust, they have limitations in terms of low signal amplification efficiency and restricted quantitative accuracy [36,37]. 25(OH)D, the major circulating form of vitamin D, is a ~400 Da lipophilic molecule with low epitope density and high protein-binding affinity, which further accentuates the limitations of conventional assay formats. To overcome these structural limitations, we established an anti-idiotype antibody-based sandwich type LFA, overcoming the structural barriers in small-molecule vitamin D detection. Also, by applying optimal buffer system, stabilizing the structure of the 25(OH)D—vitamin D-binding protein (VDBP) complex in vivo, and optimizing the dissociation of 25(OH)D from the binding protein during detection, the system achieved quantitative signal consistency and reproducibility. These results suggest that, unlike conventional assay formats, this approach provides a practical and scalable platform capable of quantitative vitamin D measurement in POCT environments. Against this background, the Vita-D Rapid Kit maintained clinical accuracy and ease of use while expanding the analysis performance of mobile-based diagnostics by combining the idiotype monoclonal antibody that recognizes the 25(OH)D-antibody binding site with algorithm-based image processing technology.

By integrating advanced image acquisition and computational analysis functions within smartphone applications, the analysis performance was further strengthened [38]. Signal variations that commonly occur in smartphone-based diagnostics are attributed to differences in lighting, optics, user manipulation, camera alignment, etc. [39]. The Vita-D Rapid Kit minimized these variations through functions such as automatic lighting control, background calibration, internal reference value normalization, and algorithm-based ROI detection [40]. The pre-calibrated regression model reduced user dependence and improved reproducibility by converting pixel intensity into three clinically meaningful categories (deficiency, insufficient, and sufficient). In addition, consistent results were achieved on both Android and iOS platforms, and high reproducibility and cross-platform reliability were secured without being affected by differences in hardware or operating systems (r = 0.99, R^2^ = 0.9967). As a result of clinical verification, the Vita-D Rapid Kit demonstrated high analytical robustness (5–100 ng/mL) and translational potential. As a result of comparison with the Atellica IM 1600 analyzer, the overall category agreement was 97.0% and Cohen’s κ coefficient was 0.951, showing high concordance (r = 0.99, R2 ≥ 0.98). The diagnostic accuracy at the clinically important deficiency interval (<20 ng/mL) was 93.3%, slightly lower than the overall concordance rate due to the limitation of the number of samples, but it performed sufficiently to identify the risk group for vitamin D related disease. In addition, the matrix equivalence test between serum and capillary blood samples showed 100% category agreement, demonstrating analytical equivalence of the minimally invasive fingerstick sampling method. These characteristics increase accessibility and patient compliance, supporting various application possibilities such as home use, resource-restricted environment, and large-scale screening tests. Integration with mobile platforms has further expanded clinical and public health values.

Nevertheless, some limitations exist. The performance of sandwich-type LFIA depends heavily on the orientation of antibodies and membrane chemical properties, and batch-to-batch variation during the manufacturing process can affect quantitative consistency near the detection limit. In addition, quality differences in antibody production arrangements, consistency of manufacturing processes, stability management of the manufacturing environment such as temperature and humidity are also important factors to maintain product reproducibility and reliability. These limitations are expected to minimize production deviations or performance heterogeneous problems that may occur during mass production by strengthening the quality control system (QC system) and increase the possibility of commercialization of the product. Although there was little cross-reaction under controlled conditions, the possibility of interference in complex matrices such as fat-soluble metabolites and VDBP according to individual differences is considered to require further verification through large-scale clinical trials. In addition, this analysis provides step-by-step vitamin D information in the form of semi-quantitative results rather than complete quantitative. In the future, further development is expected to be needed as a complete quantitative method by optimizing calibration algorithms, standardizing analysis, and securing traceability to certified reference materials. Also, performance verification in various real-world environments such as lighting, temperature, and user manipulation is essential, and data security, privacy, and regulatory compliance must also be considered for commercialization.

The vitamin D field diagnosis platform developed in this study shortens the analysis time to less than about 10 to 15 min compared to the existing automated immunoassay equipment, enabling rapid testing in clinical and home environments. In addition, the inspection process is simplified, so it is possible to perform the inspection without professional manpower, and there is little cost of installing and maintaining equipment, which can significantly reduce inspection costs. Through reading using a smartphone camera and analyzing results based on a dedicated application, users can check results in real-time and can lead to remote monitoring and medical staff counseling in connection with a cloud server or hospital computer network. This structure is suitable not only for patient monitoring in hospitals, but also for telemedicine environments such as self-testing of the elderly, chronically ill, and subjects with discomfort in behavior.

Furthermore, this platform has a modular architecture, so it can be expanded into a multi-detection health indicator management system that can analyze various biomarkers (e.g., ferritin, Calprotectin, Ca) simultaneously in the future. Therefore, this technology is expected to be used not only in clinical diagnosis but also in various fields such as personalized healthcare, telemedicine, and public health monitoring. Smartphone apps enable data collection, long-term tracking of biomarkers, and real-time data sharing with medical staff [41,42], which contributes to the implementation of personalized precision medicine. Individual supplemental therapy, continuous monitoring, and continuity of care can be supported, especially in groups such as people with limited sunlight exposure, chronically ill, and the elderly. In addition, the scalability and connectivity of smartphone-based systems have shown applicability to population-level screening tests, public health monitoring, and epidemiological analysis, leading to social values beyond individual diagnoses.

In summary, the Vita-D Rapid Kit presents a new paradigm for a small molecule-based POCT platform that combines advanced immunoassay engineering, computational analysis technology, and mobile integration technology. By overcoming the fundamental technical limitations of 25(OH)D detection and enabling reliable clinical vitamin D evaluation from minimally invasive samples, the system has led to the transition to a decentralized, patient-centered, and data-based diagnostic system. In the future, along with the continuous development of the digital health ecosystem, improvement of test sensitivity, automation of analysis, and cloud-based data integration are expected to further accelerate the development of smartphone-based diagnostic devices, which are expected to contribute greatly to the transformation of the future medical system centered on preventive medicine. This study suggested the possibility of developing a field diagnosis platform that can quickly and easily quantify vitamin D, and in the future, we plan to improve quantitative accuracy and expand it to a multi-biomarker simultaneous analysis system.

In addition, it is expected to achieve maximum usability in clinical and home diagnostic environments by automating the entire process from sample collection to results.

## Figures and Tables

**Table 1:** Evaluation of performance reproducibility and repeatability of the Vita-D Rapid Kit using standard reference materials across Android and iOS applications. Results demonstrate within-laboratory precision, between-lot reproducibility, inter-operator variability, and site-to-site consistency.

Experiment	Results	Substance	Lot 1			Total	Accordance Rate
Repeatability							
Within laboratory precision	No. of replicate/ App (Level/Result)	VITDSC-N	25/25 (1/Deficiency)			25/25 (1/Deficiency)	100%
		VITDSC-L	25/25 (1/Deficiency)			25/25 (1/Deficiency)	100%
		VITDSC-M	25/25 (2/Insufficient)			25/25 (2/Insufficient)	100%
		VITDSC-H	25/25 (3/Sufficient)			25/25 (3/Sufficient)	100%
	TOTAL		100/100			100/100	100%
**Reproducibility**							
**Experiment**	**Results**	**Substance**	**Lot 1**	**Lot 2**	**Lot 3**	**Total**	**Accordance Rate**
Between lot precision	No. of replicate/ App (Level/Result)	VITDSC-N	25/25 (1/Deficiency)	25/25 (1/Deficiency)	25/25 (1/Deficiency)	75/75 (1/Deficiency)	100%
		VITDSC-L	25/25 (1/Deficiency)	25/25 (1/Deficiency)	25/25 (1/Deficiency)	75/75 (1/Deficiency)	100%
		VITDSC-M	25/25 (2/Insufficient)	25/25 (2/Insufficient)	25/25 (2/Insufficient)	75/75 (2/Insufficient)	100%
		VITDSC-H	25/25 (3/Sufficient)	25/25 (3/Sufficient)	25/25 (3/Sufficient)	75/75 (3/Sufficient)	100%
	TOTAL		100/100	100/100	100/100	300/300	100%
**Experiment**	**Results**	**Substance**	**Lot 1**			**Total**	**Accordance Rate**
			**Operator 1**	**Operator 2**	**Operator 3**		
Between-operator precision	No. of replicate/ App (Level/Result)	VITDSC-N	25/25 (1/Deficiency)	25/25 (1/Deficiency)	25/25 (1/Deficiency)	75/75 (1/Deficiency)	100%
		VITDSC-L	25/25 (1/Deficiency)	25/25 (1/Deficiency)	25/25 (1/Deficiency)	75/75 (1/Deficiency)	100%
		VITDSC-M	25/25 (2/Insufficient)	25/25 (2/Insufficient)	25/25 (2/Insufficient)	75/75 (2/Insufficient)	100%
		VITDSC-H	25/25 (3/Sufficient)	25/25 (3/Sufficient)	25/25 (3/Sufficient)	75/75 (3/Sufficient)	100%
	TOTAL		100/100	100/100	100/100	300/300	100%
**Experiment**	**Results**	**Substance**	**LOT** **1**			**Total**	**Accordance Rate**
			**Lab 1**	**Lab 2**	**Lab 3**		
Between laboratories precision	No. of replicate/ App (Level/Result)	VITDSC-N	25/25 (1/Deficiency)	25/25 (1/Deficiency)	25/25 (1/Deficiency)	75/75 (1/Deficiency)	100%
		VITDSC-L	25/25 (1/Deficiency)	25/25 (1/Deficiency)	25/25 (1/Deficiency)	75/75 (1/Deficiency)	100%
		VITDSC-M	25/25 (2/Insufficient)	25/25 (2/Insufficient)	25/25 (2/Insufficient)	75/75 (2/Insufficient)	100%
		VITDSC-H	25/25 (3/Sufficient)	25/25 (3/Sufficient)	25/25 (3/Sufficient)	75/75 (3/Sufficient)	100%
	TOTAL		100/100	100/100	100/100	300/300	100%

**Table 2:** Correlation analysis between the Vita-D Rapid Kit and a commercial vitamin D analyzer using residual serum samples. Sample distribution categorized by vitamin D concentration, app classification level, and sex (age range: 19–88 years).

Concentration(ng/mL)	App Level	No of Samples	Sex	
			Female	Male
<10	1	2	2	0
10.0–19.9	1	13	7	6
20.0–29.9	2	39	29	10
30.0–40.9	3	9	5	4
41.0–60.9	3	24	23	1
61.0–79.9	3	11	9	2
>80.0	3	2	2	0
Total		100	77	23

**Table 3:** Correlation analysis between the Vita-D Rapid Kit and a commercial vitamin D analyzer using residual serum samples. Comparative analysis of classification outcomes between the Vita-D Rapid Kit and the Atellica IM 1600 analyzer on Android and iOS platforms.

AOS		**Atellica IM 1600 Analyzer (Atellica IM Vitamin D Total (VitD)**			Total
Vita-D Rapid Kit	Result	0~<20 ng/mL (Level 1)	20~30 ng/mL (Level 2)	>30~100 ng/mL (Level 3)	
	0~<20 ng/mL (Level 1)	14	1	0	15
	20~30 ng/mL (Level 2)	1	38	1	40
	>30~100 ng/mL (Level 3)	0	0	45	45
Total		15	39	46	100
Agreement rate (95% CI, %)		93.3% (70.2–98.8%)	97.4% (86.8–99.6%)	97.8% (88.7–99.6%)	97.0% (93.0–99.1%)
iOS		**Atellica IM 1600 Analyzer (Atellica IM Vitamin D Total (VitD)**			Total
Vita-D Rapid Kit	Result	0~<20 ng/mL (Level 1)	20~30 ng/mL (Level 2)	>30~100 ng/mL (Level 3)	
	0~<20 ng/mL (Level 1)	14	1	0	15
	20~30 ng/mL (Level 2)	1	38	1	40
	>30~100 ng/mL (Level 3)	0	0	45	45
Total		15	39	46	100
Agreement rate (95% CI, %)		93.3% (70.2–98.8%)	97.4% (86.8–99.6%)	97.8% (88.7–99.6%)	97.0% (93.0–99.1%)

**Table 4:** Equivalency assessment of specimen types using paired capillary blood and serum samples. Sample distribution categorized by vitamin D concentration, app classification level, and sex (age range: 23–44 years).

Concentration (ng/mL)	App Level	No of Samples	Sex	
			Female	Male
<10	1	1	1	0
10.0–19.9	1	11	9	2
20.0–30.0	2	7	4	3
30.1–39.9	3	2	2	0
>40.0	3	1	1	0
Total		22	17	5

**Table 5:** Equivalency assessment of specimen types using paired capillary blood and serum samples. Comparative analysis of classification concordance between the Vita-D Rapid Kit and the reference analyzer across specimen types.

AOS		**Atellica IM 1600 Analyzer** **(Atellica IM Vitamin D Total (VitD)** **/** **(Serum)**			Total
Vita-D Rapid Kit / (Capillary blood)	Result	0~<20 ng/mL (Level 1)	20~30 ng/mL (Level 2)	>30~100 ng/mL (Level 3)	
	0~<20 ng/mL (Level 1)	12	1	0	13
	20~30 ng/mL (Level 2)	0	6	0	6
	>30~100 ng/mL (Level 3)	0	0	3	3
Total		12	7	3	22
Agreement rate (95% CI, %)		100% (75.8–100%)	86% (48.7–97.4%)	100% (43.9–100%)	95.5% (78.2–99.1%)
iOS		**Atellica IM 1600 Analyzer** **(Atellica IM Vitamin D Total (VitD)** **/** **(Serum)**			Total
Vita-D Rapid Kit / (Capillary blood)	Result	0~<20 ng/mL (Level 1)	20~30 ng/mL (Level 2)	>30~100 ng/mL (Level 3)	
	0~<20 ng/mL (Level 1)	12	1	0	13
	20~30 ng/mL (Level 2)	0	6	0	6
	>30~100 ng/mL (Level 3)	0	0	3	3
Total		12	7	3	22
Agreement rate (95% CI, %)		100% (75.8–100%)	86% (48.7–97.4%)	100% (43.9–100%)	95.5% (78.2–99.1%)

## Data Availability

No new data were created or analyzed in this study. Data sharing is not applicable to this article.

## Supplementary Materials

The following supporting information can be downloaded at: https://www.mdpi.com/article/10.3390/diagnostics15222916/s1, Figure S1: Structure and components of the vitamin D lateral flow assay strip; Table S1: Comparison of various point-of-care testing (POCT) kits for Vitamin D measurement; Table S2: Standard reference materials and app interpretation criteria used for repeatability and reproducibility evaluation; Table S3; Protocol for evaluating the repeatability and reproducibility of the high-d application; Table S4: Evaluation of limit of detection (LoD) using the Vita-D Rapid Kit; Table S5: Evaluation of interference and cross-reactivity using the Vita-D Rapid Kit.

## Informed Consents Statement

Written informed consent was obtained from all subjects involved in the study. Written informed consent for publication of clinical details and/or images was obtained from the patient, or, where applicable, from a parent, guardian, or legal representative, in accordance with the International Committee of Medical Journal Editors (ICMJE) guidelines.
