# Supplementary material for: Development and Evaluation of a Smartphone App-Based Rapid 25-Hydroxy Vitamin D Test
Source: Diagnostics (Basel). 2025 Nov 18;15(22):2916. doi: 10.3390/diagnostics15222916 (PMC12651333; doi:10.3390/diagnostics15222916)
Supplement: Supplementary file 1 [file diagnostics-15-02916-s001.zip › diagnostics-3958425-supplementary.pdf]

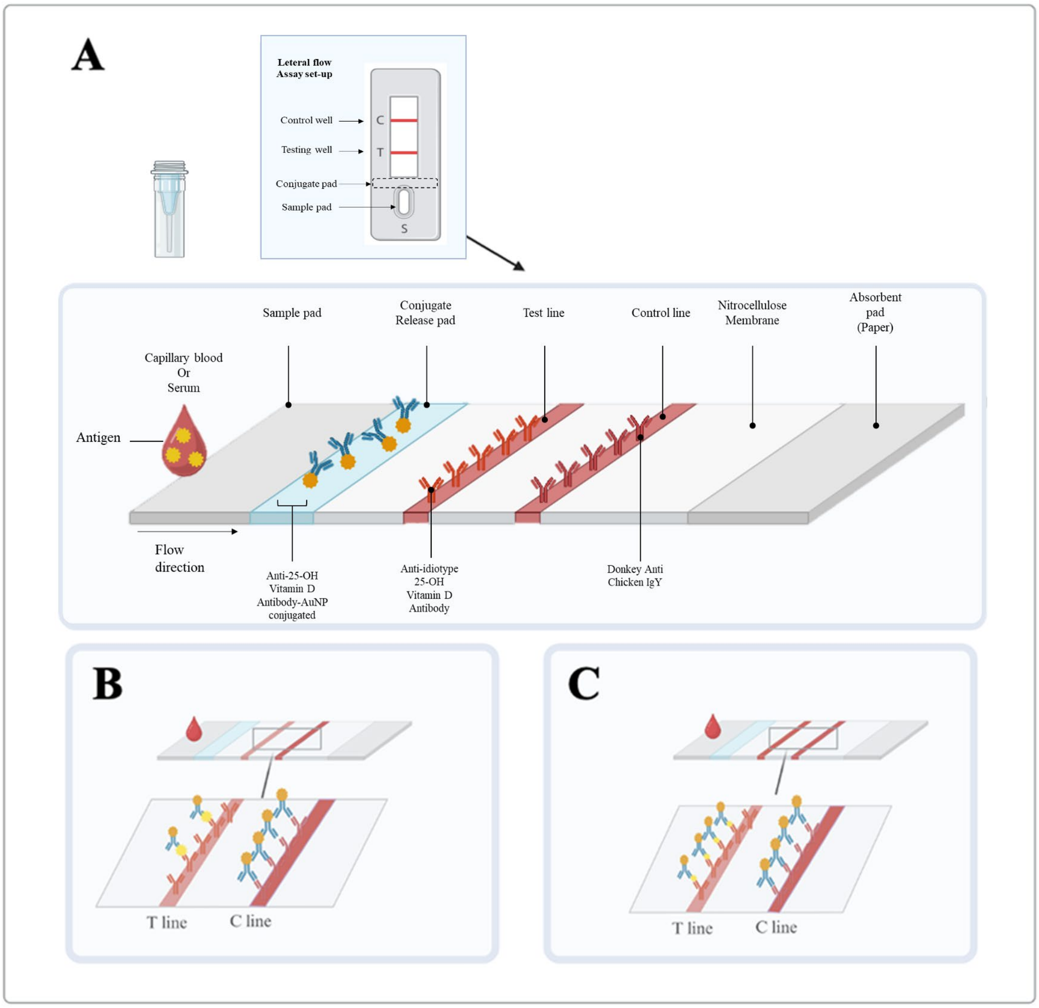

Figure S1. Structure and components of the vitamin D lateral flow assay strip. (A) Schematic representation of the sandwich-binding assay principle, showing signal generation through interaction between 25(OH)D and AuNP-conjugated antibodies in capillary blood or serum. (B) A low 25(OH)D concentration produces a faint T-line, while (C) a high concentration yields a strong signal.

Table S1. Comparison of various point-of-care testing (POCT) kits for Vitamin D measurement.

Product	Principle/ Sample Type	Range (ng/ml)	Correlation/Regression /Reference	Precision (CV, etc.)	Notes/Remarks
i-CHROMA Vitamin D (Boditech)	Fluorescent Immunoassay (FIA) / Serum, Plasma, Whole blood	~5-100	R = 0.959 (y = 0.945x + 2.9424, n = 100) / r^2≈0.70 vs ALTM	CV = 9.7-10.9% (10 ng/mL) 8–10% (50 ng/mL)	Calibration required Whole blood (30ul), serum (100ul) Measurement time ~12 min.
STANDARD F Vitamin D FIA (SD Biosensor)	Fluorescent Immunoassay (FIA) / Serum, Plasma	8-100	Y = 0.937X + 1.347, R = 0.960 (n = 100 vs ECLIA)	QCL CV = 5.3% / QCH CV = 6.3%	Sample volume 35 μL Measurement time ≈ 45 min.
Sofia Quantitative Vitamin D FIA (Quidel)	Fluorescent Immunoassay (FIA) / Serum	10-100	R = 0.908 (95% CI 0.867–0.937) vs LC-MS/MS	CV = 15.1%(12.2 ng/mL) 11.7% (21.2 ng/mL)	Sample volume 100 μL Measurement time ≈ 15 min.
AFIAS Vitamin D (Menarini)	Fluorescent Immunoassay (FIA) /Serum, Plasma, whole blood	5-100	Very good correlation reported vs LC-MS/MS	CV = 10%	Calibration required Whole blood (30ul), serum (100ul) Measurement time ~12 min.
Preventis SmarTest / SmarTest Pro Vitamin D (Preventis)	Chromatographic immunoassay (cassette) / Capillary blood	Sandwich ; Cut-Off 5-100	Correlation data not publicly available; manufacturer reports qualitative agreement with reference LC-MS/MS	Precision (CV) not specified	Home (self-test) and Pro (professional) versions available. Smartphone apps perform quantitative reading. Capillary blood (10ul) Measurement time ~15 min.
ALLTEST Vitamin D Rapid Test Cassette (Alltest)	Chromatographic immunoassay (cassette) / Capillary blood, Serum	Competitive ; Cut-off 30 -100	Manufacturer reports Agreement rate 94.0% (95%CI:87.52~97.22)	Precision (CV) not specified	Semi-quantitative visual test; provides categorical results. Color chart required Capillary blood (10ul) Measurement time ~15 min.
Vita-D Rapid kit	Chromatographic immunoassay (cassette) / Capillary blood, Serum	Sandwich ; Cut-off 5-100	In these reports R^2=0.98 (95% CI: 93.0 - 99.1) / Atellica IM 1600 Analyzer	Precision (CV) not specified	Semi-quantitative visual test; provides categorical results by Smartphone app. Capillary blood (10ul), serum (5ul) Measurement time ~15 min.

Data were collected from the manufacturer's technical documentation of each POCT kit, and instructions for use (IFU) of each POCT kit. Each instruction manual was referred to the organized product data of Boditech Med, SD Biosensor, Quidel Corp, Menarini Diagnostics, Preventis GmbH, and All Test Biotech (2018–2024).

Table S2. Standard reference materials and app interpretation criteria used for repeatability and reproducibility evaluation.

(A) Information on standard reference materials

Items		Range	Average(ng/ml)	Level	Results
Negative	Vitamin depleted serum (VITDSC-N)	0~<5	<2	1	Deficiency
Positive	VITDSC-L (DEQAS 618)	5~<20	17.2	1	Deficiency
	VITDSC-M (DEQAS 577)	20~30	24.2	2	Insufficient
	VITDSC-H (DEQAS 600)	>30~100	63.5	3	Sufficient

(B) Example of result window showing converted vitamin D concentrations

obtained from smartphone-based image analysis

	VITDSC-N	VITDSC-L	VITDSC-M	VITDSC-H	Invalid
Level	Level 1	Level 1	Level 2	Level 3	
Results	Deficiency	Deficiency	Insufficient	Sufficient	Invalid
Conc. (ng/ml)	0~<20		20~30	>30~100	(Non-appearance of the C line)
	0~<5 (Non-appearance of the T line)	5~<20 (appearance of the T line)			
Result Window	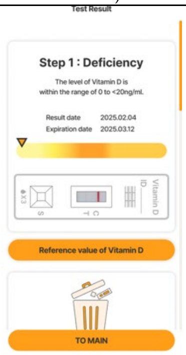	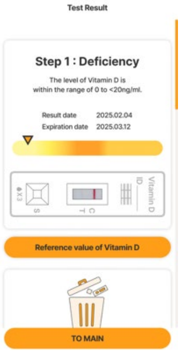	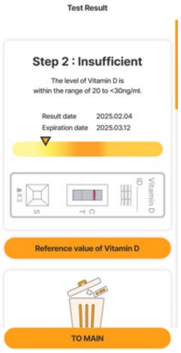	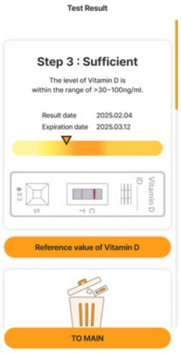	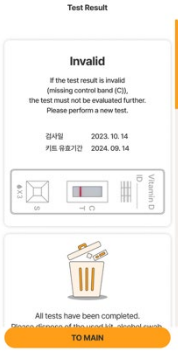

Table S3. Protocol for evaluating the repeatability and reproducibility of the high-d application. Experimental design includes within-laboratory precision, between-lot, inter-operator, and inter-laboratory reproducibility.

Experiment		Times	Lot	Operator	Testing site
Repeatability (within-laboratory precision)	1	- 5 replicate X 5 days X 2 high-d apps (Android & iOS)	Lot 1	Operator 1	Lab 1
Reproducibility (between-lot, operator, and laboratory precision)	2	- 5 replicate X 5 days X 2 high-d apps (Android & iOS)	Lot 1	Operator 1	Lab 1
			Lot 2		
			Lot 3		
	3	- 5 replicate X 5 days X 2 high-d apps (Android & iOS)	Lot 1	Operator 1	Lab 1
				Operator 2	
				Operator 3	
	4	- 5 replicate X 5 days X 2 high-d apps (Android & iOS)	Lot 1	Operator 1	Lab 1
					Lab 2
					Lab 3

Table S4. Evaluation of limit of detection (LOD) using the Vita-D Rapid Kit.

Pool No.	Dilution rate (General Control: Serum)	Atellica IM 1600 Analyzer (ng/ml)
1	10:0	> 120.0
2	8:2	108.66±0.06
3	6:4	58.70±0.06
4	5:5	41.57±0.19
5	4:6	29.78±0.08
6	2.8	7.90±0.03
7	1:9	5.20±0.01
8	0.5:9.5	< 2.0

Substance	Results	Day 1	Day 2	Day3	Day 4	Day5	Total	Accordance rate
1	Visual	+	+	+	+	+	+	Visual judgment rate 25/25 (100%) App evaluation rate 25/25 (100%)
	No. of replicate/ App (Level/Result)	5/5 (3/Sufficient)	5/5 (3/Sufficient)	5/5 (3/Sufficient)	5/5 (3/Sufficient)	5/5 (3/Sufficient)	25/25 (3/Sufficient)	
2	Visual	+	+	+	+	+	+	Visual judgment rate 25/25 (100%) App evaluation rate 25/25 (100%)
	No. of replicate/ App (Level/Result)	5/5 (3/Sufficient)	5/5 (3/Sufficient)	5/5 (3/Sufficient)	5/5 (3/Sufficient)	5/5 (3/Sufficient)	25/25 (3/Sufficient)	
3	Visual	+	+	+	+	+	+	Visual judgment rate 25/25 (100%) App evaluation rate 25/25 (100%)
	No. of replicate/ App (Level/Result)	5/5 (3/Sufficient)	5/5 (3/Sufficient)	5/5 (3/Sufficient)	5/5 (3/Sufficient)	5/5 (3/Sufficient)	25/25 (3/Sufficient)	
4	Visual	+	+	+	+	+	+	Visual judgment rate 25/25 (100%) App evaluation rate 25/25 (100%)
	No. of replicate/ App (Level/Result)	5/5 (3/Sufficient)	5/5 (3/Sufficient)	5/5 (3/Sufficient)	5/5 (3/Sufficient)	5/5 (3/Sufficient)	25/25 (3/Sufficient)	
5	Visual	+	+	+	+	+	+	Visual judgment rate 25/25 (100%) App evaluation rate 25/25 (100%)
	No. of replicate/ App (Level/Result)	5/5 (2/Insufficient)	5/5 (2/Insufficient)	5/5 (2/Insufficient)	5/5 (2/Insufficient)	5/5 (2/Insufficient)	25/25 (2/Insufficient)	
6	Visual	+	+	+	+	+	+	Visual judgment rate 25/25 (100%) App evaluation rate 25/25 (100%)
	No. of replicate/ App (Level/Result)	5/5 (1/Deficiency)	5/5 (1/Deficiency)	5/5 (1/Deficiency)	5/5 (1/Deficiency)	5/5 (1/Deficiency)	25/25 (1/Deficiency)	
7	Visual	+	+	+	+	+	+	Visual judgment rate 25/25 (100%) App evaluation rate 25/25 (100%)
	No. of replicate/ App (Level/Result)	5/5 (1/Deficiency)	5/5 (1/Deficiency)	5/5 (1/Deficiency)	5/5 (1/Deficiency)	5/5 (1/Deficiency)	25/25 (1/Deficiency)	
8	Visual	-	-	-	-	-	-	Visual judgment rate 25/25 (100%) App evaluation rate 25/25 (100%)
	No. of replicate/ App (Level/Result)	5/5 (1/Deficiency)	5/5 (1/Deficiency)	5/5 (1/Deficiency)	5/5 (1/Deficiency)	5/5 (1/Deficiency)	25/25 (1/Deficiency)	

Substance	Results	Day 1	Day 2	Day3	Total	Accordance rate
6	Visual	+	+	+	+	Visual judgment rate 60/60 (100%) App evaluation rate 60/60 (100%)
	No. of replicate/ App (Level/Result)	20/20 (1/Deficiency)	20/20 (1/Deficiency)	20/20 (1/Deficiency)	60/60 (1/Deficiency)	
7	Visual	+	+	+	+	Visual judgment rate 60/60 (100%) App evaluation rate 60/60 (100%)
	No. of replicate/ App (Level/Result)	20/20 (1/Deficiency)	20/20 (1/Deficiency)	20/20 (1/Deficiency)	60/60 (1/Deficiency)	
8	Visual	-	-	-	-	Visual judgment rate 60/60 (100%) App evaluation rate 60/60 (100%)
	No. of replicate/ App (Level/Result)	20/20 (1/Deficiency)	20/20 (1/Deficiency)	20/20 (1/Deficiency)	60/60 (1/Deficiency)	

Table S5. Evaluation of Interference and Cross-Reactivity Using the Vita-D Rapid Kit.

(A) The Vita-D Rapid Kit showed no evidence of analytical interference from any of the tested substances.

No.	Interference substance	#Cat/Manufacture	Concentration
1	Aspirin	#PHR1003, Sigma	0.2mg/ml
2	Atropine	#A-046, Sigma	0.2mg/ml
3	Bilirubin Conjugate	#201102, Sigma	0.3mg/ml
4	Bilirubin	#B4126, Sigma	0.3mg/ml
5	Cholesterol	#C8667, Sigma	10mg/ml
6	Gentisic acid	#G5129, Sigma	0.2mg/ml
7	D-(+)-Glucose	#G7021, Sigma	1mg/ml
8	Intralipid	#I141, Sigma	3mg/ml
9	Triglyceride	#17810, Sigma	15mg/ml
10	Human Serum Albumin	#SRP6182, Sigma	120mg/ml
11	Biotin	#14400, Sigma	6mg/ml
12	Caffein	#C0750, Sigma	0.2mg/ml
13	Human Hemoglobin	#H7379, Sigma	2mg/ml
14	Acetaminophen	#A7085, Sigma	0.2mg/ml

App (Level/Result)						
**Substance**	**Results**	**VITDSC-N**	**VITDSC-L**	**VITDSC--M**	**VITDSC-H**	**Accordance rate**
Absence of interfering substances	No. of replicate/ App (Level/Result)	3/3 (1/Deficiency)	3/3 (1/Deficiency)	3/3 (2/Insufficien t)	3/3 (3/Sufficient)	VITDSC-N: 3/3(100%) VITDSC-L: 3/3(100%) VITDSC-M: 3/3(100%) VITDSC-H: 3/3 (100%)
Aspirin	No. of replicate/ App (Level/Result)	3/3 (1/Deficiency)	3/3 (1/Deficiency)	3/3 (2/Insufficien t)	3/3 (3/Sufficient)	VITDSC-N: 3/3(100%) VITDSC-L: 3/3(100%) VITDSC-M: 3/3(100%) VITDSC-H: 3/3 (100%)
Atropine	No. of	3/3	3/3	3/3	3/3	VITDSC-N: 3/3(100%)

	replicate/ App (Level/Result)	(1/Deficiency)	(1/Deficiency)	(2/Insufficient)	(3/Sufficient)	VITDSC-L: 3/3(100%)<br>VITDSC-M: 3/3(100%)<br>VITDSC-H: 3/3 (100%)
Bilirubin Conjugate	No. of replicate/ App (Level/Result)	3/3 (1/Deficiency)	3/3 (1/Deficiency)	3/3 (2/Insufficient)	3/3 (3/Sufficient)	VITDSC-N: 3/3(100%)<br>VITDSC-L: 3/3(100%)<br>VITDSC-M: 3/3(100%)<br>VITDSC-H: 3/3 (100%)
Bilirubin	No. of replicate/ App (Level/Result)	3/3 (1/Deficiency)	3/3 (1/Deficiency)	3/3 (2/Insufficient)	3/3 (3/Sufficient)	VITDSC-N: 3/3(100%)<br>VITDSC-L: 3/3(100%)<br>VITDSC-M: 3/3(100%)<br>VITDSC-H: 3/3 (100%)
Cholesterol	No. of replicate/ App (Level/Result)	3/3 (1/Deficiency)	3/3 (1/Deficiency)	3/3 (2/Insufficient)	3/3 (3/Sufficient)	VITDSC-N: 3/3(100%)<br>VITDSC-L: 3/3(100%)<br>VITDSC-M: 3/3(100%)<br>VITDSC-H: 3/3 (100%)
Gentisic acid	No. of replicate/ App (Level/Result)	3/3 (1/Deficiency)	3/3 (1/Deficiency)	3/3 (2/Insufficient)	3/3 (3/Sufficient)	VITDSC-N: 3/3(100%)<br>VITDSC-L: 3/3(100%)<br>VITDSC-M: 3/3(100%)<br>VITDSC-H: 3/3 (100%)
D-(+) Glucose	No. of replicate/ App (Level/Result)	3/3 (1/Deficiency)	3/3 (1/Deficiency)	3/3 (2/Insufficient)	3/3 (3/Sufficient)	VITDSC-N: 3/3(100%)<br>VITDSC-L: 3/3(100%)<br>VITDSC-M: 3/3(100%)<br>VITDSC-H: 3/3 (100%)
Intralipid	No. of replicate/ App (Level/Result)	3/3 (1/Deficiency)	3/3 (1/Deficiency)	3/3 (2/Insufficient)	3/3 (3/Sufficient)	VITDSC-N: 3/3(100%)<br>VITDSC-L: 3/3(100%)<br>VITDSC-M: 3/3(100%)<br>VITDSC-H: 3/3 (100%)
Triglyceride	No. of replicate/ App (Level/Result)	3/3 (1/Deficiency)	3/3 (1/Deficiency)	3/3 (2/Insufficient)	3/3 (3/Sufficient)	VITDSC-N: 3/3(100%)<br>VITDSC-L: 3/3(100%)<br>VITDSC-M: 3/3(100%)<br>VITDSC-H: 3/3 (100%)
Human Serum Albumin	No. of replicate/ App (Level/Result)	3/3 (1/Deficiency)	3/3 (1/Deficiency)	3/3 (2/Insufficient)	3/3 (3/Sufficient)	VITDSC-N: 3/3(100%)<br>VITDSC-L: 3/3(100%)<br>VITDSC-M: 3/3(100%)<br>VITDSC-H: 3/3 (100%)
Biotin	No. of replicate/ App (Level/Result)	3/3 (1/Deficiency)	3/3 (1/Deficiency)	3/3 (2/Insufficient)	3/3 (3/Sufficient)	VITDSC-N: 3/3(100%)<br>VITDSC-L: 3/3(100%)<br>VITDSC-M: 3/3(100%)<br>VITDSC-H: 3/3 (100%)
Caffein	No. of replicate/ App (Level/Result)	3/3 (1/Deficiency)	3/3 (1/Deficiency)	3/3 (2/Insufficient)	3/3 (3/Sufficient)	VITDSC-N: 3/3(100%)<br>VITDSC-L: 3/3(100%)<br>VITDSC-M: 3/3(100%)<br>VITDSC-H: 3/3 (100%)
Human Hemoglobin	No. of replicate/ App (Level/Result)	3/3 (1/Deficiency)	3/3 (1/Deficiency)	3/3 (2/Insufficient)	3/3 (3/Sufficient)	VITDSC-N: 3/3(100%)<br>VITDSC-L: 3/3(100%)<br>VITDSC-M: 3/3(100%)<br>VITDSC-H: 3/3 (100%)
Acet-aminophen	No. of replicate/ App (Level/Result)	3/3 (1/Deficiency)	3/3 (1/Deficiency)	3/3 (2/Insufficient)	3/3 (3/Sufficient)	VITDSC-N: 3/3(100%)<br>VITDSC-L: 3/3(100%)<br>VITDSC-M: 3/3(100%)<br>VITDSC-H: 3/3 (100%)

No.	Cross-reaction Substance	#Cat/Manufacture	Concentration
1	1α,25-Dihydroxyvitamin D2	#17944, Sigma	100ng/ml
2	1α,25-Dihydroxyvitamin D3	#17936, Sigma	100ng/ml
3	25-Hydroxyvitamin D2	#17937, Sigma	100ng/ml
4	25-Hydroxyvitamin D3	#H-083, Sigma	100ng/ml
5	Vitamin A (Retinol)	#7632, Sigma	16mg/L

6	Vitamin B12	#2876, Sigma	25mg/L
7	L-Ascorbic acid (Vitamin C)	#A92902, Sigma	2500mg/L
8	Vitamin D2 solution	#740217, Sigma	1,000ng/ml
9	Vitamin D3 solution	#740292, Sigma	1,000ng/ml
10	α-Tocopherol (Vitamin E)	#V-020, Sigma	20mg/L
11	Vitamin K1	#V3501, Sigma	0.5mg/L
12	Folic acid (Vitamin M)	#F7876, Sigma	1mg/L

(B) The Vita-D Rapid Kit exhibited no detectable cross-reactivity with any of the evaluated substances.

App (Level/Result)						
**Substance**	**Results**	**VITDSC-N**	**VITDSC-L**	**VITDSC--M**	**VITDSC-H**	**Accordance rate**
Absence of cross reaction substances	No. of replicate/ App (Level/Result)	3/3 (1/Deficiency)	3/3 (1/Deficiency)	3/3 (2/Insufficien t)	3/3 (3/Sufficient)	VITDSC-N: 3/3(100%) VITDSC-L: 3/3(100%) VITDSC-M: 3/3(100%) VITDSC-H: 3/3 (100%)
1α,25-Dihydroxyvita min D2	No. of replicate/ App (Level/Result)	3/3 (1/Deficiency)	3/3 (1/Deficiency)	3/3 (2/Insufficien t)	3/3 (3/Sufficient)	VITDSC-N: 3/3(100%) VITDSC-L: 3/3(100%) VITDSC-M: 3/3(100%) VITDSC-H: 3/3 (100%)
1α,25-Dihydroxyvita min D3	No. of replicate/ App (Level/Result)	3/3 (1/Deficiency)	3/3 (1/Deficiency)	3/3 (2/Insufficien t)	3/3 (3/Sufficient)	VITDSC-N: 3/3(100%) VITDSC-L: 3/3(100%) VITDSC-M: 3/3(100%) VITDSC-H: 3/3 (100%)
25-Hydroxyvitami n D2	No. of replicate/ App (Level/Result)	3/3 (1/Deficiency)	3/3 (1/Deficiency)	3/3 (2/Insufficien t)	3/3 (3/Sufficient)	VITDSC-N: 3/3(100%) VITDSC-L: 3/3(100%) VITDSC-M: 3/3(100%) VITDSC-H: 3/3 (100%)
25-Hydroxyvitami n D3	No. of replicate/ App (Level/Result)	3/3 (1/Deficiency)	3/3 (1/Deficiency)	3/3 (2/Insufficien t)	3/3 (3/Sufficient)	VITDSC-N: 3/3(100%) VITDSC-L: 3/3(100%) VITDSC-M: 3/3(100%) VITDSC-H: 3/3 (100%)
Vitamin A (Retinol)	No. of replicate/ App (Level/Result)	3/3 (1/Deficiency)	3/3 (1/Deficiency)	3/3 (2/Insufficien t)	3/3 (3/Sufficient)	VITDSC-N: 3/3(100%) VITDSC-L: 3/3(100%) VITDSC-M: 3/3(100%) VITDSC-H: 3/3 (100%)
Vitamin B12	No. of replicate/ App (Level/Result)	3/3 (1/Deficiency)	3/3 (1/Deficiency)	3/3 (2/Insufficien t)	3/3 (3/Sufficient)	VITDSC-N: 3/3(100%) VITDSC-L: 3/3(100%) VITDSC-M: 3/3(100%) VITDSC-H: 3/3 (100%)
L-Ascorbic acid (Vitamin C)	No. of replicate/ App (Level/Result)	3/3 (1/Deficiency)	3/3 (1/Deficiency)	3/3 (2/Insufficien t)	3/3 (3/Sufficient)	VITDSC-N: 3/3(100%) VITDSC-L: 3/3(100%) VITDSC-M: 3/3(100%) VITDSC-H: 3/3 (100%)
Vitamin D2 solution	No. of replicate/ App (Level/Result)	3/3 (1/Deficiency)	3/3 (1/Deficiency)	3/3 (2/Insufficien t)	3/3 (3/Sufficient)	VITDSC-N: 3/3(100%) VITDSC-L: 3/3(100%) VITDSC-M: 3/3(100%) VITDSC-H: 3/3 (100%)
Vitamin D3 solution	No. of replicate/ App (Level/Result)	3/3 (1/Deficiency)	3/3 (1/Deficiency)	3/3 (2/Insufficien t)	3/3 (3/Sufficient)	VITDSC-N: 3/3(100%) VITDSC-L: 3/3(100%) VITDSC-M: 3/3(100%) VITDSC-H: 3/3 (100%)
α-Tocopherol (Vitamin E)	No. of replicate/	3/3 (1/Deficiency	3/3 (1/Deficiency	3/3 (2/Insufficien	3/3 (3/Sufficient)	VITDSC-N: 3/3(100%) VITDSC-L: 3/3(100%)

	App (Level/Result)	)	)	t)		VITDSC-M: 3/3(100%)<br>VITDSC-H: 3/3 (100%)
Vitamin K1	No. of replicate/ App (Level/Result)	3/3 (1/Deficiency)	3/3 (1/Deficiency)	3/3 (2/Insufficien t)	3/3 (3/Sufficient)	VITDSC-N: 3/3(100%)<br>VITDSC-L: 3/3(100%)<br>VITDSC-M: 3/3(100%)<br>VITDSC-H: 3/3 (100%)
Folic acid (Vitamin M)	No. of replicate/ App (Level/Result)	3/3 (1/Deficiency)	3/3 (1/Deficiency)	3/3 (2/Insufficien t)	3/3 (3/Sufficient)	VITDSC-N: 3/3(100%)<br>VITDSC-L: 3/3(100%)<br>VITDSC-M: 3/3(100%)<br>VITDSC-H: 3/3 (100%)
